# Family Violence Among Older Adult Patients Consulting in Primary Care Clinics: Results From the ESA (Enquête sur la santé des aînés) Services Study on Mental Health and Aging

**DOI:** 10.1177/070674371405900805

**Published:** 2014-08

**Authors:** Michel Préville, Samia Djemaa Mechakra-Tahiri, Helen-Maria Vasiliadis, Véronique Mathieu, Louise Quesnel, Samantha Gontijo-Guerra, Catherine Lamoureux-Lamarche, Djamal Berbiche

**Affiliations:** 1 Professor, Faculty of Medicine and Health Sciences, Université de Sherbrooke, Sherbrooke, Quebec; Researcher, Research Center, Charles Le Moyne Hospital, Greenfield Park, Quebec.; 2 Researcher, Enquête sur la santé des aînés Research Program, Charles Le Moyne Hospital, Greenfield Park, Quebec.; 3 Professor, Faculty of Medicine and Health Sciences, Université de Sherbrooke, Sherbrooke, Quebec; Researcher, Research Center, Charles Le Moyne Hospital, Greenfield Park, Quebec.; 4 Doctoral Candidate, Department of Psychology, Université du Québec en Outaouais, Gatineau, Quebec.; 5 Family Physician, Centre Médical Saint Hubert, Saint Hubert, Quebec; Chief of Regional Department of General Medicine of Montérégie, Longueuil, Quebec.; 6 Student, Clinical Sciences Program, Faculty of Medicine and Health Sciences, Université de Sherbrooke, Sherbrooke, Quebec.; 7 Senior Statistician, Research Center, Charles Le Moyne Hospital, Greenfield Park, Quebec.

**Keywords:** family violence, older adults, populational study

## Abstract

**Objective:**

To document the reliability and construct validity of the Family Violence Scale (FVS) in the older adult population aged 65 years and older.

**Method:**

Data came from a cross-sectional survey, the Enquête sur la santé des aînés et l’utilisation des services de santé (ESA Services Study), conducted in 2011–2013 using a probabilistic sample of older adults waiting for medical services in primary care clinics (*n* = 1765). Family violence was defined as a latent variable, coming from a spouse and from children.

**Results:**

A model with 2 indicators of violence; that is, psychological and financial violence, and physical violence, adequately fitted the observed data. The reliability of the FVS was 0.95. According to our results, 16% of older adults reported experiencing some form of family violence in the past 12 months of their interview, and 3% reported a high level of family violence (FVS > 0.36). Our results showed that the victim’s sex was not associated with the degree of violence (β = 0.02). However, the victim’s age was associated with family violence (β = −0.12). Older adults, aged 75 years and older, reported less violence than those aged between 65 and 74 years.

**Conclusion:**

Our results lead us to conclude that family violence against older adults is common and warrants greater public health and political attention. General practitioners could play an active role in the detection of violence among older adults.

## Abstract (fr)

**Objectif:**

Documenter la validité et la fiabilité de l’échelle de violence familiale (EVF) dans la population âgée de 65 ans et plus.

**Méthode:**

Les données proviennent de l’étude transversale ESA-services (Enquête sur la santé des aînés et l’utilisation des services de santé), réalisée de 2011 à 2013 à l’aide d’un échantillon probabiliste de personnes âgées en attente de services médicaux dans des cliniques de soins de première ligne (*n* = 1765). La violence familiale a été définie comme une variable latente, déterminée par deux sources : le conjoint et les enfants.

**Résultats:**

Nos résultats ont montré qu’un modèle spécifiant deux indicateurs pour chaque source de violence, soit la violence psychologique et financière et la violence physique, correspondait adéquatement aux données observées. La fiabilité de l’EVF était de 0,95. Selon nos résultats, 16 % des personnes âgées ont rapporté de la violence familiale et 3 % avaient un niveau élevé de violence familiale (EVF > 0,36). Nos résultats ont montré que le sexe de la victime n’était pas lié (β = 0,02) au degré de violence, mais que l’âge de la victime était associé à la violence familiale (β = −0,12). Les personnes âgées de 75 ans et plus ont rapporté moins de violence que celles âgées de 65 à 74 ans.

**Conclusion:**

Nos résultats suggèrent que la violence familiale contre les personnes âgées est fréquente et mérite une plus grande attention politique et de la santé publique. Également, les médecins généralistes pourraient jouer un rôle actif dans la détection de la violence chez les patients âgés.

Family violence is a major public health problem, with important physical and mental health consequences for the victims. In Canada, since 1980, it is a crime.^1^ Family violence in the elderly is also an important problem because of its association with psychological distress,^2^,^3^ an increased use of health services^4^ and a high risk of mortality.^5^,^6^ However, few studies have also considered the issue among older women and men.^3^,^7^–^16^

The prevalence of family violence in older adults varies from one study to another. These inconsistencies may be related to differences in the sociocultural context of the populations studied or to methodological differences regarding the definition and measurement of family violence,^3^,^17^–^20^ which make it difficult to interpret results.^4^

In addition, methods of data collection can introduce information bias associated with the social desirability phenomenon. As, for example, in the case where data are collected from police registers.^21^ This source of information may cause an underestimation of the prevalence of family violence in older adults, who are often reluctant to report violence they have experienced, fearing reprisals.^22^ In fact, 1 out of 14 incidents are reported to the police.^23^ Data on family violence obtained from telephone interviews or self-administered questionnaires may also be subject to both selection and information bias, which can all impact prevalence estimates.^11^,^18^,^24^

Population surveys reported that between 3% and 22% of older adults suffered from at least 1 form of family violence in the past year.^3^,^6^,^9^,^10^,^17^,^23^,^25^ In Canada, few data exist on the prevalence of family violence against older adults.^8^,^11^,^26^ A study conducted in 1993 by Podnieks^11^ showed that 4% of Canadians were victims of at least 1 form of violence, often at the hands of family members or a loved one.

The different types of elder abuse include psychological abuse, the most commonly reported, followed by physical abuse, financial, exploitation, and neglect.^11^,^23^,^27^–^29^

Factors associated with family violence among older adults are multiple and vary depending on the population studied and the type of violence committed.^30^ Women would be more often victims of violence than men.^8^,^6^,^18^ Regarding age, studies have not reported consistent findings. Some research has shown that older adults, aged 75 years and older, are more at risk of being victims of violence,^31^ while other studies suggested no association between age and the likelihood of being a victim of violence.^10^,^28^

In Canada, the police report on family violence^26^ indicated that older adults, aged between 65 to 74 years, are more likely to be victims of violence than those aged 75 years and older. Men appear to suffer more from physical abuse than women, although the latter would be more exposed to violence that could lead to medical consultation or hospitalization.^32^ Other factors, such as ethnicity (being not Caucasian),^33^ language barriers,^24^,^32^ poverty,^34^ ageism,^32^ functional disability,^6^,^8^,^33^,^35^ alcoholism,^36^,^37^ social isolation, and low social support,^6^,^23^,^38^ are also considered to be risk factors of family violence in the older adult population.

**Clinical Implications**

- Our study provided evidence-based data on the prevalence of family violence in the elderly population waiting for health services in primary health services clinics.
- Our study documented the validity and reliability of the FVS which could be used in the elderly population waiting for health services in primary health services clinics.
- Our study gave estimates of unmet needs in the elderly population in primary health services clinics.

**Limitations**

- We used self-reported information from respondents.
- Clinical validity of the FVS is limited.
- Our sample was limited to the Quebec population.

Family violence may affect many aspects of the victim’s life (physical, economic, psychological, and social).^5^,^6^,^39^ According to some studies, the risk of psychological distress and suicide has been shown to be higher among victims of family violence.^1^,^39^,^40^ Family violence has also been associated with increased risk of injury requiring medical treatment and hospitalization.^3^,^4^,^32^,^40^

The objective of our study was to document the reliability and construct validity of the FVS used in the ESA services research program and to document its association with age and sex in the older adult population, aged 65 years and older. Based on the FVS psychometric properties, we reported the prevalence of family violence among community-dwelling older adults waiting for medical services in primary health clinics.

## Conceptual Framework

**Figure 1:**
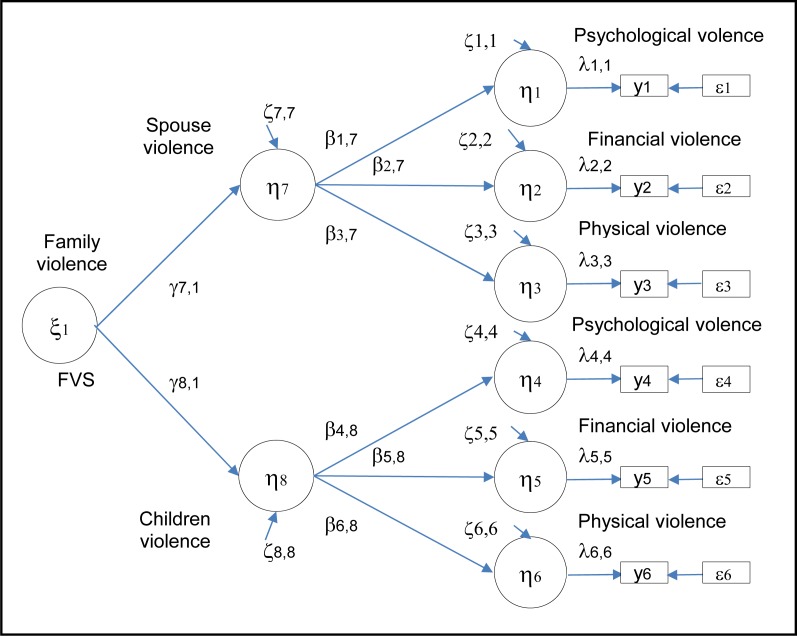
**Conceptual model of family violence measurement in older adults (FVS = Family Violence Scale)**

In the ESA services research program, family violence was defined as a latent variable, determined by violence from a spouse and violence from one or more children. Three main sources of violence were considered: psychological, financial, and physical. This measurement model of family violence can be represented using Figure 1.

In this model, the 6 indicators of violence (y_i_) (i = 1, 2, 3, 4, 5, 6) are observed variables considered to be measured without error as their reliability is unknown. The validity coefficients of psychological, financial, and physical spousal violence indicators (λ_i,j_) (i = 1, 2, 3) and children violence indicators (i = 4, 5, 6) and the error (ɛ_i_) terms were, therefore, set to 1 and 0, respectively. Betas (β_j,j_) are the regression coefficients between the psychological, financial, and physical violence latent variables (η_j_) (j = 1, 2, 3, 4, 5, 6), and the spouse (η_j_) (j = 7) and children violence (η_j_) (j = 8) latent variables. Gammas (γ) are the regression coefficients representing the association between the construct of family violence (ξ_1_) and the spouse (η_7_) and children (η_8_) component of family violence. Zetas (ζ_j_) (j = 7,8) represent the variance of the latent variables of family violence attributable to spouse (ζ_7_) and children (ζ_8_) explained by external causes not measured in our study. Zetas (ζ_j_) (j = 1, 2, 3, 4, 5, 6) represent the variance of the latent variables not explained by the constructs of spouse and children violence.

## Method

Data used in our study came from a cross-sectional survey, the ESA Services Study, conducted from 2011 to 2013 using a probabilistic sample of older adults, aged 65 years and older, waiting for medical services in primary health clinics in 1 of the health regions of Quebec. The health and social services agency taking part in this study is responsible for a population of 1 325 000 inhabitants.

A sample of GPs working full-time with their main practice in the territory of the collaborative health agency was constituted. The sampling plan of the study included stratification according to 4 types of primary medical health services organizations:

1. the family medicine group,
2. local community health services centres,
3. PCs with less than 3 GPs (PCs < 3), and
4. PCs with at least 3 GPs (PCs ≥ 3).

On a list of 838 physicians, 744 were eligible. Among the latter, 409 agreed to participate in the study but 245 physicians effectively recruited patients. An average of 7.3 voluntary patients per participating physician was subsequently included in the study. The participation rate of GPs in the study was 33%.

Data were weighted to ensure that the true proportions of older adult patients in each type of primary medical health services organizations were reflected in the analysis. Weights were determined based on the following:

1. the probability of participation of the types of primary medical health services organizations [π(a)];
2. the conditional probability of participation of the physicians in each type of primary medical health services organizations [π(b/a)], and
3. the conditional probability of participation of the patient in the physician medical health clinic [π(c/ab)].

## Procedure

Patients aged 65 years and older who visited 1 of the participating physicians during the study period received, in the clinic’s waiting room, a pamphlet describing the objectives and the length of the study and inviting them to participate in a face-to-face interview at home. The volunteers had to leave a phone number where they could be reached and had to complete the K10, a short screening questionnaire about depression^41^ prior to the consultation with their doctor, who was not aware of the patient’s results on the K10. Patients were subsequently reached by phone by the study coordinator within 30 days to book an appointment. A compensation of Can$15 was provided to the participants to ensure a sufficient participation rate. This project received approval from the Ethics Committee of the Charles LeMoyne Hospital. In total, 1811 patients agreed to participate in the interview at home.

Interviewers were health professionals (*n* = 19) who received a 1-day training session on the administration of the computerized ESA questionnaire. At the beginning of the interview, lasting on average 90 minutes, written consent was obtained from the respondents to carry out the interview. To avoid desirability and information bias associated with the presence of another family member or friend, the interviews were conducted in the most isolated area of the house possible.

As memory problems affect the accuracy of the information given, patients with a moderate or severe cognitive problem, based on the Mini-Mental State Examination (<22) (*n* = 46), were excluded at the beginning of the interview. Then, patients without cognitive problems were invited to answer the ESA questionnaire concerning their physical and mental health status and individual predisposing and contextual facilitating factors of the use of health services. The weighted sample included 1765 elderly patients.

## Measures

In our study, family violence was measured using an adaptation of the CTS2. The CTS2 includes 78 items and 5 subscales (negotiation, psychological aggression, physical assault, injury, and sexual coercion). Reliability and construct validity of the CTS2 have previously been reported.^20^,^42^

The ESA FVS contains 21 questions from the CTS2, including 4 items measuring spousal psychological violence, 4 items measuring financial violence, and 3 items measuring physical violence. It also includes 4 items measuring children psychological violence, 4 items measuring financial violence, and 2 items measuring physical violence (online eAppendix). Respondents were asked to answer yes or no to the 21 questions of the FVS questionnaire using a numeric keypad. To limit potential social desirability bias, the answers given by respondents were unreadable by the interviewer. Respondents were previously informed of this confidentiality procedure. Three variables measuring the frequency of the psychological, financial, and physical violence events in the past 12 months preceding the interview were constructed. Owing to the asymmetric distribution of these indicators, the values of these variables were grouped on an ordered scale ranging from 0 to 2 events and more.

## Analyses

The construct validity of the FVS was tested using LISREL, version 8.80.^43^ As the variables were not normally distributed, the polychoric correlations matrix and the variances and covariances asymptotic matrix were used to estimate the parameters of the hypothetical model (Figure 1). The maximum likelihood robust chi-square statistic, the RMSEA index, was used to guide the overall evaluation of the models. We also used the chi-square and degrees of freedom ratio, which reflects how many times the observed chi-square value is greater than its expected value. A ratio of less than 3 indicates a satisfactory fit. The RMSEA index evaluates the error of approximation of the model in the population. This index varies from 0 to 1. A value of less than 0.05 indicates a satisfactory adjustment. We used the 95% threshold of statistical significance for our analyses. Finally, a comparison group strategy was used to test our hypothesis about the invariance of the measurement model of family violence according to the victims’ age and sex.^44^ The H statistic was used to document the reliability of FVS. This measure indicates the average total variance of the items taken into account by the latent construct.^45^ Finally, the association of the FVS with the respondents’ age and sex was examined. The age was grouped in 2 categories: from 65 to 74 years, and 74 years and older.

## Results

The mean age of respondents was 73.4 years (SD 6.1), and 57.3% were women. Our results showed that 16% (*n* = 282) of older adults who consulted in the general medical sector reported family violence in the past 12 months (Table 1).

More specifically, results showed that 9.9% of older adults in the past 12 months suffered psychological violence from their spouse, among whom 1.1% also suffered financial violence and 1.2% suffered physical violence during this period. Results also indicated that 7.3% of older adults suffered psychological violence from their children, among whom 1.5% suffered financial violence and 1% suffered physical violence during the last 12 months. Almost 9% (25/282) of older adults reported both sources (spouse and children) of violence.

### The Measurement Model

**Figure 2:**
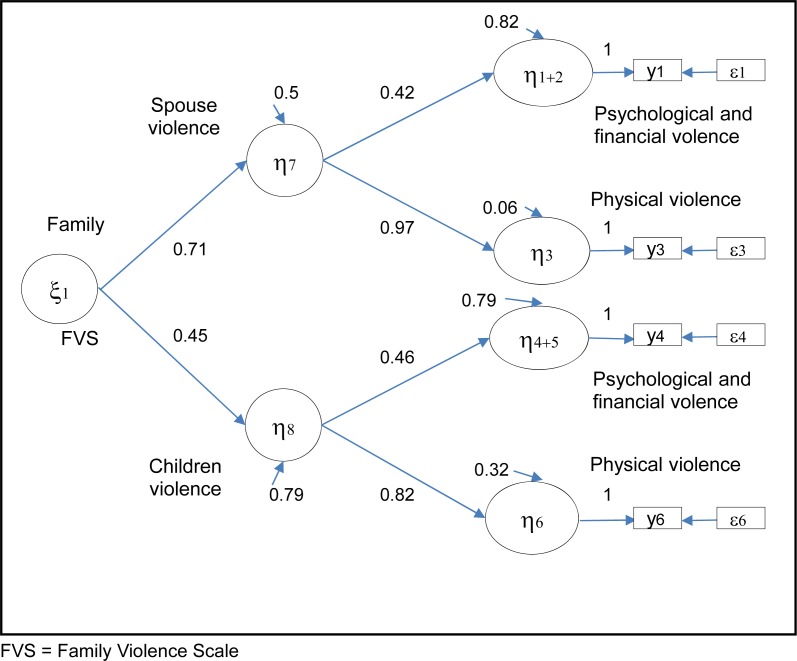
**Respecified measurement model of family violence in older adults (standardized coefficients)**

Results showed that a measurement model of family violence (M_1_), including the 2 dimensions of violence from a spouse and from children, and each source measured by 3 indicators—psychological, financial, and physical violence—did not adequately fit the observed data (χ^2^ = 37.31, *df* = 8, *P* < 0.001; RMSEA = 0.045). Based on the modification indices values obtained from the factor analysis of model M_1_, financial violence was integrated into the psychological violence dimension. A second model (M_2_), with only 2 indicators per dimension—psychological and financial violence—and physical violence was tested (Figure 2). This model fit adequately the observed data (χ^2^ = 2.32, *df* = 1, *P* = 0.13, RSMEA = 0.03). The internal consistency reliability coefficient calculated for the FVS was 0.95.

In this model, the latent variable representing the degree of violence from the spouse (η_7_) explained 94% of the variance of the physical violence (η_3_) reported by older adults and 18% of the variance of the psychological violence (η_1+2_). Results also indicated that physical violence, although less common, was a more important determinant (2.31; 0.97/0.42) of the degree of violence from the spouse than psychological violence. Similar results were observed regarding family violence from children (η_8_). This latent variable explained 68% of the variance of physical violence reported by older adults (η_6_) and 21% of the variance of psychological violence (η_4+5_). Physical violence was also a more important determinant 1.78 (0.82/0.46) of the degree of violence from children than psychological violence.

Results showed that a measurement model specifying invariance of the factor structure of the family violence FVS scale, according to a victims’ sex (χ^2^ = 15.36, *df* = 11, *P* = 0.17; RMSEA = 0.021) and age (χ^2^ = 8.96, *df* = 11, *P* = 0.63; RMSEA = 0.000), was plausible. In addition, our results also indicated that violence from a spouse was moderately (φ = 0.32) associated with violence from children. When the analysis was restricted to the sample of older adults married and having children, our results did not show a stronger association (φ = 0.40) between these 2 sources of violence.

The degree of violence experienced by older adults as measured by the FVS scale ranged from 0.00 to 1.51. This factorial score represents the score that would have reported older adults if it would have been possible to measure the latent variable of family violence directly without error. Results indicated that subjects with a score equal to or higher than 0.36 (*n* = 52) had a score greater than the 1 corresponding to the 80th percentile of the distribution of older adults who reported family violence and represent 3% of the older adult population.

**Figure 3:**
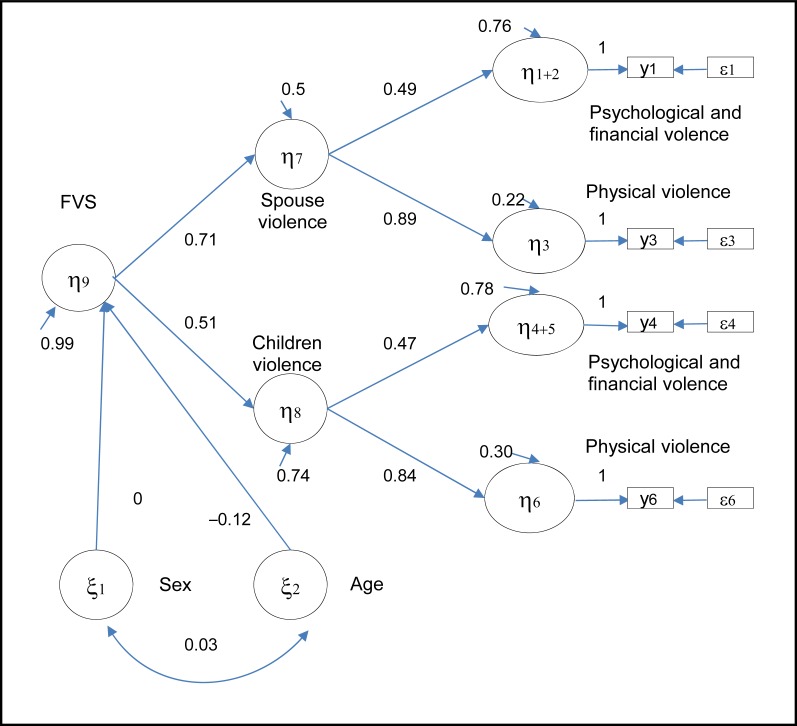
**Regression model of the Family Violence Scale (FVS) on sex and age in older adults**

Finally, as Figure 3 indicates, a victim’s sex was not significantly associated with the degree of violence (β = 0.00). Results also showed that older adults aged 75 years and older faced less violence than those aged between 65 and 74 years (β = −0.12).

## Discussion

Results showed that a measurement model of family violence consisting of 2 dimensions, violence from a spouse and violence from children, adequately fit the observed data in both age and sex groups. The internal consistency reliability coefficient calculated for the FVS was 0.95, suggesting adequate reproducibility of the results obtained with the FVS.

Based on our results, 16% of older adults who consulted in the general medical sector reported family violence in the past 12 months. In addition, our results indicated that 3% of older adults reported a high level of family violence (FVS > 0.36) in the past 12 months. These results are concordant with those reported in other studies.^9^,^3^,^10^,^11^,^17^,^46^

Our results also showed that 9.9% of older adults suffered psychological violence from their spouse in the past 12 months, including 1.1% of financial violence, and that 1.2% of older adults suffered physical violence during this period. These results are similar to those reported in other studies.^3^,^23^,^28^ Further, our results showed a similar distribution of the different types of violence from a spouse and children.

Results also showed that physical violence was a more important determinant of the level of violence than psychological violence, as well from a spouse than from children. This result is consistent with the idea that physical violence is at the one end of the gradient of severity of family violence. To our knowledge, until now, no study has compared the different types of violence carried out by spouses and children of older adults.

Our results indicated that violence from a spouse is moderately (φ = 0.32) associated with violence from children. This relatively low association hardly supports the hypothesis of a family transmission of violence^47^ but is consistent with results from studies reporting that family violence is common among people who have been exposed to parental violence during their childhood.^48^,^49^

In our study, a victim’s sex was not significantly associated with the degree of violence (β = 0.00). Results from other studies on family violence and sex are inconsistent.^10^,^25^,^28^,^29^ Regarding age, our results showed that a victim’s age was associated with family violence (β = −0.12). In our study, older adults, aged 75 years and older, reported enduring less violence than those aged between 65 and 74 years. Results from other studies on family violence and age are also inconsistent.^6^,^10^,^26^,^28^,^31^ These differences in the literature may be attributed to variations in the definitions of family violence, survey methodology, and chosen age group categories.

Finally, our results should be interpreted while taking into account certain limitations. First, the use of different definitions of family violence and the inclusion of multiple categories of aggressors limits the comparison of our results with those reported in other studies. Second, our results are also limited to the population living at home, having a family doctor, able to participate in an interview at home, and to volunteer to answer a questionnaire on family violence. Third, given that memory problems would affect the accuracy of the information returned, older adults with moderate or severe cognitive problems were excluded from our study at the beginning of the interview. This strategy may have introduced a selection bias that may have resulted in an underestimation of the true prevalence of family violence in the older adult population. Fourth, given that the recall period of the violent events was 12 months (as in several studies), recall bias may be present and could have impacted our results. Although our methodology included the option of participants personally logging in their answers to the family violence module, a social desirability bias may have influenced the declaration of violent events experienced by older adults. All these factors may have impacted the estimation of the true prevalence of family violence in the older adult population.

Despite these limitations, our results were obtained from a large sample of elderly people living at home in Quebec who consult in the general medical sector. They were obtained through face-to-face interviews carried out at-home by trained interviewers using a standardized interviewing procedure designed to minimize information and social desirability bias and to maximize the validity of the information obtained from respondents. Our study provided information on the validity and reliability of a family violence measurement (FVS), easy to administer, well accepted by older adults, that can be used to estimate prevalence of psychological, financial, and physical family violence among older adults.

## Conclusion

Our results showed that 16% of older adults who consulted in the general medical sector reported family violence in the past 12 months and that 3% of these older adults reported a high level of family violence. This accounted for almost 208 146 and 39 000 older adults, aged 65 years and older, in Quebec in 2012. Our results also showed an association between family violence from a spouse and violence from children. These results lead us to conclude that family violence perpetrated against older adults warrants greater public health and political attention.

It has been previously reported that GPs do not take into account family violence during their consultations with their older patients.^50^ The high prevalence of family violence observed in our study underlines the need for increased awareness and need for training in the detection of family violence in older adult patients where GPs could play an active role in the detection of violence among older adults.

## 



## Figures and Tables

**Table 1:** Characteristics of respondents

Characteristic	Sample (*n* = 1765) *n* (%)
Sex	
Women	1011 (57.3)
Men	754 (42.7)
Age, years	
65 to 74	1103 (62.5)
≥75	662 (37.5)
Presence of family violence from spouse	176 (10.0)
Presence of family violence from children	131 (7.4)

## Abbreviations

CTS2: Conflict Tactics Scale—Revised
ESA: Enquête sur la santé des aînés
FVS: Family Violence Scale
GP: general practitioner
K10: Kessler 10-Item Psychological Distress Scale
PC: private clinic
RMSEA: root mean square error of approximation
