# Supplementary material for: Family Violence Among Older Adult Patients Consulting in Primary Care Clinics: Results From the ESA (Enquête sur la santé des aînés) Services Study on Mental Health and Aging
Source: Can J Psychiatry. 2014 Aug;59(8):426–33. doi: 10.1177/070674371405900805 (PMC4143299; doi:10.1177/070674371405900805)
Supplement: Supplementary file 1 [file CJP-2014-vol59-August-426-433-eAppendix.pdf]

Préville M, Mechakra-Tahiri SD, Vasiliadis H-M, et al. Family violence among older adult patients consulting in primary care clinics: results from the ESA (Enquête sur la santé des aînés) Services Study on Mental Health and Aging. Can J Psychiatry. 2014;59(8):426–433.

eAppendix Échelle de violence familiale (EVF)/ Family violence Scale (FVS)

Maintenant, j'aimerais vous poser des questions sur vos relations avec votre entourage. Dites-moi si les énoncés suivants s'appliquent à vous?
I would like to now ask you questions about your relationship with your members of your familyl. Tell me if the following statements apply to you?

1 Au cours des 12 derniers mois, mon(ma) conjoint(e) m'a insulté... lors d'une dispute avec moi.
In the past 12 months, my spouse insulted me... during a dispute we had.

2 Au cours des 12 derniers mois, mon (ma) conjoint(te) m'a poussé ou frappé à cause d'une dispute avec moi.

In the past 12 months, my spouse pushed or hit me during an altercation.

3 Au cours des 12 derniers mois, mon(ma) conjoint(e) a détruit quelque chose m'appartenant, à cause d'une dispute avec moi.
In the past 12 months, my spouse destroyed something belonging to me, because of a dispute we had.

4 Au cours des 12 derniers mois, j'ai eu besoin de voir un docteur à cause d'une dispute avec mon (ma) conjoint(e).
In the past 12 months, I needed to see a doctor because of a dispute I had with my spouse .

5 Au cours des 12 derniers mois, mon(ma) conjoint(e) a utilisé la force pour avoir une relation sexuelle avec moi.

In the past 12 months, my spouse used force to have sex with me.

6 Au cours des 12 derniers mois, un de mes enfants m'a insulté lors d'une dispute avec moi.
In the past 12 months, one of my children insulted me during a dispute we had.

7 Au cours des 12 derniers mois, un de mes enfants m'a poussé ou frappé à cause d'une dispute avec moi.

In the past 12 months, one of my children pushed or hit me during an altercation.

8 Au cours des 12 derniers mois, un de mes enfants a détruit quelque chose m'appartenant, à cause d'une dispute avec moi.
In the past 12 months, one of my children destroyed something belonging to me, because of a dispute we had.

9 Au cours des 12 derniers mois, j'ai eu besoin de voir un docteur à cause d'une dispute avec un de mes enfants.

In the past 12 months, I needed to see a doctor because of a dispute with one of my children.

10 Au cours des 12 derniers mois, mon(ma) conjoint(te) a essayé de limiter les contacts que j'entretient avec ma famille ou mes amis.
In the past 12 months, my spouse tried to limit my contacts with my family or my friends.

11 Au cours des 12 derniers mois, mon(ma) conjoint(e) cherchait à me rabaisser ou utilisait des mots blessants.

In the past 12 months, my spouse tried to disgrace me or used hurtful words.

12 Au cours des 12 derniers mois, mon(ma) conjoint(e) m'a empêché de connaître mon revenu, ou d'y avoir accès, même si vous le demandiez.
In the past 12 months, my spouse kept me from knowing my income, or to have access to it, even when I asked.

13 Au cours des 12 derniers mois, mon(ma) conjoint(e) insistait pour que je lui laisse la gestion de mes finances alors que je ne voulais pas.
In the past 12 months, against my wishes, my spouse insisted that I leave them my finances.

14 Au cours des 12 derniers mois, mon(ma) conjoint(e) insistait pour que je modifie mon testament alors que je ne voulais pas.
In the past 12 months, against my wishes, my spouse insisted that I change my will.

15 Au cours des 12 derniers mois, mon(ma) conjoint(e) insistait pour obtenir une procuration alors que je ne le voulais pas.
In the past 12 months, against my wishes, my spouse insisted that I give them power of attorney.

16 Au cours des 12 derniers mois, un de mes enfants a essayé de limiter les contacts que j'entretiens avec ma famille ou mes amis.
In the past 12 months, one of my children tried to limit the contact I have with my family or my friends.

17 Au cours des 12 derniers mois, un de mes enfants cherchait à me rabaisser ou utilisait des mots blessants.

In the past 12 months, one of my children tried to disgrace me or used hurtful words.

18 Au cours des 12 derniers mois, un de mes enfants vous a empêché de connaître mon revenu, ou d'y avoir accès, même si vous le demandiez.
In the past 12 months, one of my children kept me from knowing my income, or to have access to it, even when I asked.

19 Au cours des 12 derniers mois, un de mes enfants insistait pour que je lui laisse la gestion de mes finances alors que je ne voulais pas.
In the past 12 months, against my wishes,one of my children insisted that I leave them my finances.

20 Au cours des 12 derniers mois, un de mes enfants insistait pour que je modifie mon testament alors que je ne voulais pas.
In the past 12 months, against my wishes, one of my children insisted that I change my will.

21 Au cours des 12 derniers mois, un de mes enfants insistait pour obtenir une procuration alors que je ne le voulais pas.
In the past 12 months, against my wishes, one of my children insisted I give them power of attorney.
